# Effect of high-intensity interval training and moderate-intensity continuous training on blood lactate clearance after high-intensity test in adult men

**DOI:** 10.3389/fphys.2024.1451464

**Published:** 2024-09-04

**Authors:** Han Xie, Xiaojin Mao, Zhaohong Wang

**Affiliations:** ^1^ College of Physical Education and Sports, Beijing Normal University, Beijing, China; ^2^ College of Physical Education, Shandong Normal University, Jinan, China

**Keywords:** HIIT, MICT, blood lactate, adult men, train

## Abstract

This study compared the effects of High-intensity interval training (HIIT) and moderate-intensity continuous training (MICT) on blood lactate clearance. 21 adult males were equally and randomly assigned to the HIIT and MICT groups, and completed 8 weeks of training. Before the training intervention, after 4 weeks and 8 weeks of training, all subjects were tested for blood lactate levels between 0 and 55 min after the same high-intensity test. The results show that after 8 weeks, blood lactate levels were significantly lower than pre-tests in both the HIIT and MICT groups at “0–55 min” after high-intensity test (*p* < 0.05), and the blood lactate clearance percentage at15-min and 30-min in both groups were significantly higher than the pre-tests (*P* < 0.01). The blood lactate levels in the HIIT group were significantly lower than those in the MICT group at 15 min and 30 min after test (*P* < 0.05), and the blood lactate clearance percentage at 30 min in the HIIT group was significantly higher than those in the MICT group (*P* < 0.05). In conclusion, both HIIT and MICT enhance blood lactate clearance in adult males post high-intensity test, with HIIT demonstrating superior effectiveness, making it a viable alternative to MICT.

## 1 Introduction

Lactic acid is a metabolic product of glycolysis for body energy supply and is rapidly dissociated into lactate and protons (Robergs et al., 2004). The body contains a small amount of lactate at rest, whereas during high-intensity exercise (Astrand et al., 1963) or cardiorespiratory dysfunction (Mungan et al., 2018), the body is in a state of relative hypoxia and lactate concentrations increase dramatically (Brooks, 2018). The metabolic clearance of lactate is essential for improving the acid‒base balance and maintaining internal environmental homeostasis and the glycolytic energy supply rate (Juel, 1996). When lactate accumulates in large quantities, it lowers cellular and blood pH and inhibits glycolysis, which in turn affects exercise performance. The generated lactate is mainly removed by the liver, skeletal muscle, cardiac muscle cells, kidney cells, and other tissues and organs through oxidative decomposition, gluconeogenesis, the synthesis of other substances in the liver, sweating, and urination (Brooks et al., 2022).

Studies have noted that athletes have greater lactate clearance capacity than the sedentary general population (Hinojosa et al., 2021), proving that exercise is an effective way to improve the body’s lactate clearance capacity. Related studies have shown that the increased aerobic capacity associated with endurance exercise significantly improves the body’s lactate clearance capacity (Messonnier et al., 2013; Emhoff and Messonnier, 2023). Moderate-intensity continuous training (MICT) is a traditional method of aerobic capacity training that involves 30–60 min of moderate-intensity aerobic exercise (65%–75% of maximum heart rate) (Locatelli et al., 2024), and has been demonstrated to enhance the aerobic capacity of the body (Weston KS. et al., 2014). High-intensity interval training (HIIT) is a type of interval training involving multiple rounds of high-intensity exercise or sprint interval training (≥80% of maximum heart rate) (Weston M. et al., 2014) interspersed with recovery periods (MacInnis and Gibala, 2017) and has effects similar to those of strength training or endurance training (Hwang et al., 2019). As two distinct training methods for improving aerobic capacity, there is ongoing debate regarding which method is more effective in enhancing aerobic capacity and post-exercise blood lactate clearance. In terms of promoting aerobic capacity, the prevailing view suggests that HIIT is more effective than MICT, as high-intensity exercise elicits a greater physiological response (Gorostiaga et al., 1991; Schjerve et al., 2008; Martins et al., 2016; Su et al., 2019). Conversely, another perspective posits that both methods yield similar effects (Gibala et al., 2006; Cocks et al., 2015). For instance, Cock’s (2015) 4-week study found no significant difference in maximal oxygen uptake improvements between HIIT and MICT interventions. Regarding blood lactate clearance, existing research has demonstrated that HIIT can effectively enhance the body’s ability to clear blood lactate (Bishop et al., 2008); however, no studies have yet compared the intervention effects of HIIT and MICT directly. Therefore, this study aims to compare the effects of 8 weeks of HIIT and MICT interventions on blood lactate clearance capacity, providing guidance for the public and athletes in achieving optimal load management and recovery strategies in their training. We hypothesized that both HIIT and MICT contribute to the improvement of blood lactate clearance at rest after high-intensity test, but the effect of HIIT may be better.

## 2 Materials and methods

### 2.1 Subjects

The subjects of this study were 22 male college students at Beijing Normal University who were not majoring in physical education. These students were randomly assigned by computer to the MICT and HIIT groups, with 11 people in each group. All subjects were in good health, with no acute or chronic diseases; no smoking or drinking habits; no recent history of medication; no serious sports injuries within 1 year; and no previous sports training experience. During the experiment, subjects were asked to maintain a normal routine and not to participate in additional physical activities. One subject dropped out of the experiment because they failed to meet the training requirements, and finally leaving 10 in the HIIT (Age:21.56 ± 1.35 years, Height:177.30 ± 4.42 cm, Weight:71.18 ± 7.01 kg) and 11 in the MICT (Age: 20.95 ± 1.24 years, Height: 178.07 ± 4.76cm, Weight: 72.03 ± 6.31 kg) groups. The study was approved by the Ethics Committee of Beijing Normal University, and the subjects were informed of the purpose and procedure of this experiment and signed an informed consent form before the intervention.

### 2.2 Interventions

In this study, subjects in the HIIT group performed a 30-s sprint at 85%–95% HRmax, with a 3-min brisk walking or jogging between sets (Weston KS. et al., 2014), before the exercise, the subjects were instructed to run at maximum speed to achieve the specified heart rate. Subjects in the MICT group ran aerobically at an even pace at 65%–75% HRmax (Locatelli et al., 2024), and the maximum heart rate was estimated as “220 - age”. To adhere to the principle of progressive overload in exercise training, we required that energy expenditure be approximately 240 kcal during the first 3 weeks (with the HIIT group performing about six to seven sets and the MICT group engaging in about 30 min of exercise), and increase to 300 kcal during the subsequent 5 weeks (with the HIIT group performing about 8-9 sets and the MICT group engaging in about 35–40 min of exercise). This approach was implemented to mitigate the risk of inadequate adaptation to exercise volume or intensity at the onset of the training. Heart rate monitoring was conducted using the Polar H10 (Polar Electro Oy, Kempele, Finland), which has been proven to effectively track heart rate at both rest and during exercise (Schaffarczyk et al., 2022), ensuring that the intended exercise intensity was achieved. Energy expenditure was measured using the wGT3X-BT (ActiGraph, Pensacola, USA), a device commonly employed for assessing physical activity (Mielke et al., 2022); it was secured to the subject’s waist during exercise and removed at the conclusion of the session. Both the HIIT and MICT groups participated in the intervention for 8 weeks, 3 times a week, on Mondays, Wednesdays, and Fridays at 7 a.m. In addition, all subjects were asked to maintain a normal diet (prohibition of ketogenic diet, alcohol consumption, *etc.*) during the training period and were asked to keep a daily record to monitor their diet for inspection. Blood lactate clearance was tested in all subjects before the first training (pre-test), after 4 weeks of training (mid-test), and after 8 weeks of training (post-test). High-intensity test was used as a means of inducing blood lactate accumulation. To ensure the repeatability of the high-intensity test in the pre-test, mid-test and post-test, we specified that it consisted of a 1-min run at 20 km/h on a running platform with a 0-grade incline. Subjects were supervised throughout exercise. Vital capacity and body fat percentage were tested in only pre-test and post-test, and all tests were performed in the morning on an empty stomach to avoid any influence of diet on the test results (Forbes et al., 2020). Vital capacity was assessed using an electronic spirometer SF-1 (Jinyi, Jiangsu, China) (Guohua, 2001). The subjects inhaled deeply and then exhaled all their breath into the mouthpiece, ensuring there was no leakage, the test was performed three times, and the highest was taken. Body fat percentage was measured with the bioimpedance scale InBody 270 (Biospace, California USA), ensuring that subjects were in a fasted state. To reduce bias in the assessment of the results, the two groups of subjects were mixed and performed high-intensity test together and then quietly rested to recover after the exercise. Finger peripheral blood (approx. 0.5 μL) was collected using a lactate analyzer (lactate scout, EKF Diagnostics, Cardiff, UK) immediately after test (0 min) and during the recovery period at 5 min, 15 min, 30 min, 45 min and 55 min to determine the blood lactate values at different time points, and consider the highest blood lactate value measured at several time points from 0 to 55 min as the peak value. The lactate scout (EKF Diagnostics, Cardiff, UK) is a widely utilized tool for measuring blood lactate levels (Wang J et al., 2019). It was calibrated before testing, and the fingertips were disinfected with alcohol prior to blood collection. For sampling, a disposable blood collection needle was used to puncture the subject’s sterilized finger, with the first drop of blood discarded and the second drop used for testing. Blood lactate clearance percentage at each time point was calculated as the percentage of the peak lactate value, using the formula: LA _Time_%= (LA _peak_-LA _Time_)/(LA _peak_- LA _rest_) × 100%, where LA _peak_ is the peak lactate value, LA _Time_ is the lactate value at a specific time point, and LA _rest_ is the lactate value at rest.

### 2.3 Statistical analysis

In this study, after excluding data from subjects who dropped out, the remaining data were tested for normality using SPSS 25.0 (Shapiro‒Wilk). Two-way repeated measures analysis of variance (ANOVA) was used to explore changes in blood lactate at pre-test, mid-test, and post-test and the interaction effect of intervention cycle with intervention, and when an interaction effect was present, simple effects analyses were used to explore between-group differences. The differences in vital capacity and body fat percentage before and after the experiment were compared using ANOVA. All data are reported as the mean ± SD, and differences were considered significant at *p* < 0.05.

## 3 Results

### 3.1 Baseline indicators

**FIGURE 1:**
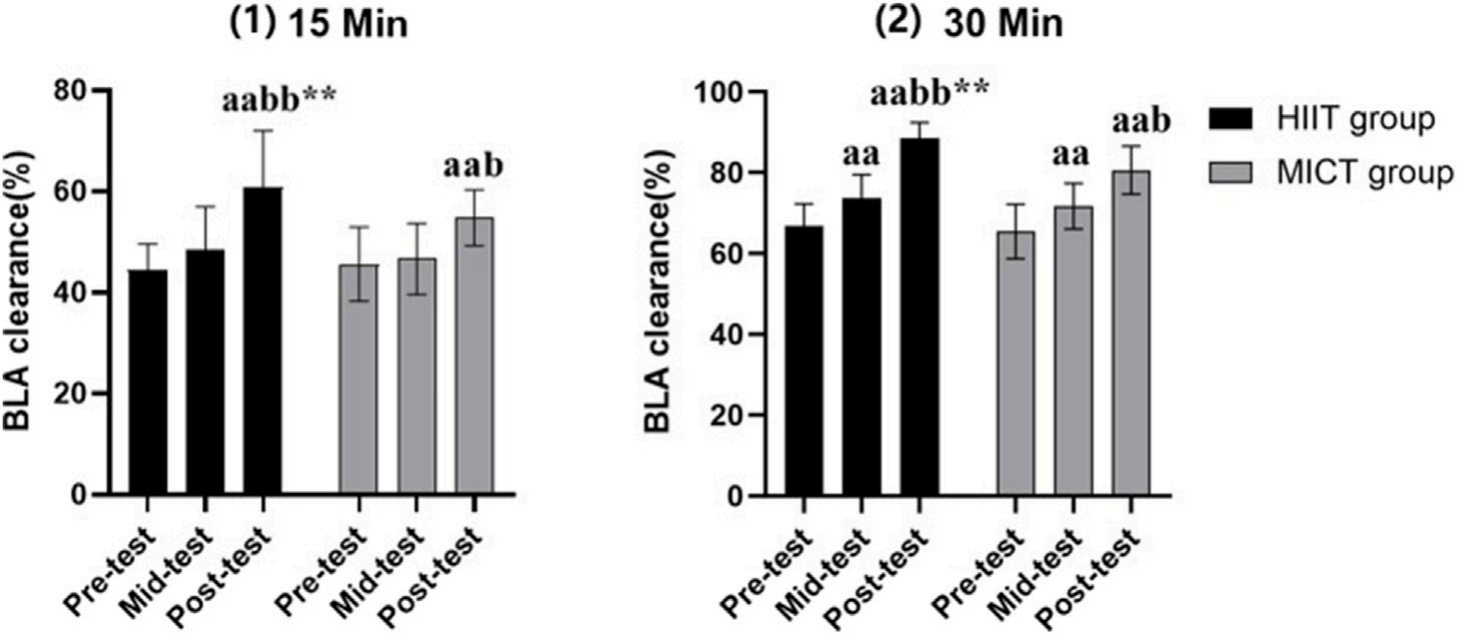
Blood lactate clearance. Compared to pre-test: a indicates *p* < 0.05, a indicates *p* < 0.01; Compared to mid-test: b indicates *p* < 0.05, bb indicates *p* < 0.01; HIIT group compared to MICT group in the same test, *indicates *p* < 0.05, ** indicates *p* < 0.01.

**FIGURE 2:**
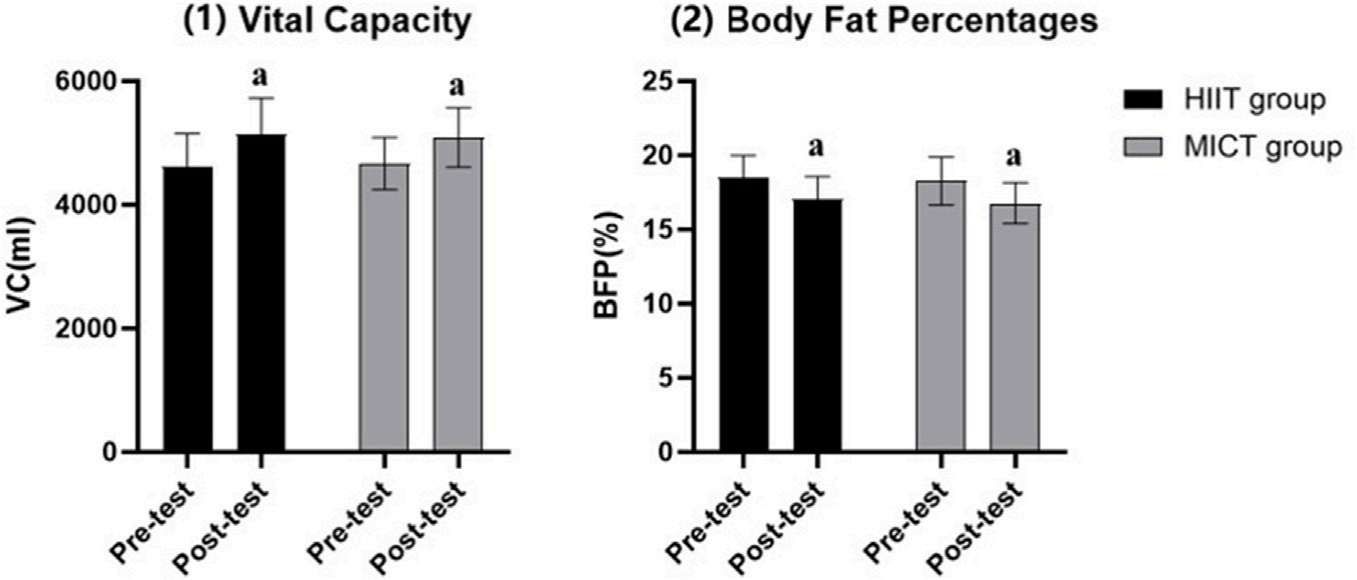
Other indicators. Compared to pre-test: a indicates *P* <0.05.

The pre-test results showed that there was no significant difference in blood lactate concentration, vital capacity and body fat percentages between the HIIT and MICT groups at any measurement point. Therefore, subjects in the HIIT and MICT groups had the same basic conditions (see Table 1; Figures 1, 2).

**TABLE 1:** Blood lactate concentrations in the HIIT and MICT groups after high-intensity test.

	HIIT			MICT		
Time	Pre-test (mmol/L)	Mid-test (mmol/L)	Post-test (mmol/L)	Pre-test (mmol/L)	Mid-test (mmol/L)	Post-test (mmol/L)
Rest	1.91 ± 0.20	1.86 ± 0.16	1.85 ± 0.16	1.97 ± 0.16	1.91 ± 0.13	1.92 ± 0.12
Peak	14.16 ± 1.79	13.35 ± 1.00^aa^	11.81 ± 0.84^aabb^	14.65 ± 0.92	13.41 ± 0.95^aa^	12.10 ± 1.13^aabb^
0 min	14.09 ± 1.75	13.20 ± 1.23^aa^	11.70 ± 0.86^aabb^	13.98 ± 0.90	13.16 ± 1.04^aa^	11.48 ± 0.75^aabb^
5 min	12.88 ± 1.78	11.37 ± 0.68^a^	10.19 ± 1.19^aabb^	13.21 ± 1.68	11.61 ± 1.92^aa^	10.83 ± 2.05^aab^
15 min	8.70 ± 1.20	7.73 ± 0.85^aa^	5.69 ± 0.85^aabb*^	8.90 ± 1.21	8.06 ± 1.11^a^	6.55 ± 0.90^aabb^
30 min	6.39 ± 1.08	4.97 ± 0.81^aa^	2.98 ± 0.43^aabb**^	6.31 ± 0.81	5.13 ± 0.84^aa^	3.93 ± 0.71^aabb^
45 min	3.74 ± 0.70	2.82 ± 0.46^aa^	2.03 ± 0.20^aabb^	3.84 ± 0.79	2.91 ± 0.58^aa^	2.25 ± 0.30^aabb^
55 min	2.40 ± 0.49	2.01 ± 0.31^aa^	1.90 ± 0.20^aa^	2.47 ± 0.47	2.07 ± 0.26^aa^	1.96 ± 0.10^aa^

Compared to pre-test: a indicates *P*< 0.05, aa indicates *P* < 0.01; Compared to mid-test: b indicates *P* < 0.05, bb indicates *P* < 0.01; HIIT, group compared to MICT, group in the same test, *indicates *p* < 0.05, ** indicates *p* < 0.01.

### 3.2 Recovery period

The post-test results showed that blood lactate concentrations at 15 min, 30 min after test were significantly different between the HIIT and MICT groups (*p* < 0.05). Additionally, in both the HIIT and MICT groups, the post-test and mid-test blood lactate concentrations were significantly lower than the pre-test concentrations immediately to 55 min after test (*p* < 0.05). The post-test blood lactate concentrations were significantly lower than the mid-test concentrations immediately to 45 min after test (*p* < 0.05) (see Table 1).

### 3.3 Peak blood lactate

In both groups, post-test values were significantly lower than in mid-test and pre-test (*p* < 0.05). In tandem, mid-test was significantly lower than the pre-test (*p* < 0.05) (see Table 1).

### 3.4 Blood lactate clearance

Due to relatively high blood lactate values at 5 min after test (with some subjects reaching peak values at this time), and the fact that blood lactate values at 45 and 55 min after test were close to resting values (with some subjects nearing full recovery), this study did not calculate the blood lactate clearance percentage at these specific time points. Despite this, there was a significant difference in the percentage of blood lactate clearance at 15 min and 30 min between HIIT (15-min: 0.64 ± 0.10; 30-min: 0.89 ± 0.04) and MICT (15-min: 0.54 ± 0.04; 30-min: 0.81 ± 0.06) at post-test (*p* < 0.01). Additionally, the blood lactate clearance at 15 min and 30 min post-test in both groups was significantly higher than the pre-test (15-min HIIT:0.44 ± 0.05; 15-min MICT: 0.45 ± 0.06; 30-min HIIT: 0.67 ± 0.05; 30-min MICT: 0.65 ± 0.07) and mid-test (15-min HIIT: 0.49 ± 0.09; 15-min MICT: 0.46 ± 0.06; 30-min HIIT: 0.74 ± 0.06; 30-min MICT: 0.72 ± 0.06) (*p* < 0.05). Also, in both the HIIT and MICT groups, the 30-min blood lactate clearance in mid-test were higher than the pre-test (*p* < 0.01) (see Figure 1).

### 3.5 Other indicators

In terms of vital capacity and body fat percentage, the post-test (HIIT: 5147.80 ± 581.13mL/17.01% ± 1.57%; MICT: 5090.18 ± 482.51mL/16.77% ± 1.38%) of both the HIIT and MICT groups were significantly better than the pre-test (HIIT: 4595.60 ± 552.13mL/18.46% ± 1.53%; MICT: 4666.00 ± 423.53mL/18.27% ± 1.62%) (*p* < 0.05) (see Figure 2).

## 4 Discussion

Currently, managing exercise training load is central to sports science practice (West et al., 2021). This study provides new insights into optimizing training strategies for enhancing performance and recovery by comparing the effects of two training methods on blood lactate clearance. The results demonstrate a significant improvement in blood lactate clearance for both training conditions, with HIIT showing a more pronounced effect. This highlights the advantage of HIIT in enhancing blood lactate clearance capabilities. Additionally, this training method may also be beneficial in clinical rehabilitation, as improving lactate clearance through exercise could potentially reduce the severity of cardiovascular diseases (Li et al., 2023) or respiratory conditions (Carpenè et al., 2021).

As shown in Table 1, blood lactate concentrations in subjects generally remained at a relatively high level from immediately after the test to 5min after. Some subjects showed a “rising-declining” curve during the recovery phase (blood lactate concentration at 5 min is higher than at 0 min), which may be attributed to the delayed diffusion of muscle lactate into the blood. After lactate is produced in large quantities at the skeletal muscle site, it has to pass through the myocyte membrane into the blood to raise the blood lactate concentration. The timing of this diffusion into the blood may influence the observed changes in blood lactate levels during the recovery process (Juel and Halestrap, 1999). Since the myocyte membrane has a limiting function on the transport of lactate and the speed of transport is related to membrane permeability (Sun et al., 2017), there may be a certain time lag in the dynamic balance of the two concentrations.

The change in blood lactate concentration is influenced by several factors, including the rate of lactate production in skeletal muscle, lactate entry into the bloodstream, and lactate clearance. In our study, we attempted to interpret the results from these three perspectives. Although we were unable to separately measure lactate production and entry, we were able to indirectly assess the combined effects of these factors on lactate concentration by measuring blood lactate levels at different time points. It should be noted that our experiment primarily assessed the overall concentration changes in lactate, rather than the specific rates of production and entry. Therefore, when interpreting the results, it is essential to consider the limitations associated with these measurements.

In this study, subjects in both the HIIT and MICT groups had significantly lower blood lactate values at all time points after the intervention. Tomlin and Wenger (2001) noted that individuals with a high aerobic capacity are less dependent on anaerobic energy supply systems and are thus able to reduce the proportion of anaerobic glycolysis during exercise. It has been previously demonstrated that HIIT and MICT have a beneficial effect on the aerobic capacity of the body (MacInnis and Gibala, 2017); therefore, it can be assumed that long-term HIIT and MICT reduce the proportion of anaerobic glycolysis during a single bout of high-intensity test, which is also confirmed by the significantly lower post-test peak blood lactate than pre-test peak blood lactate observed in this study. The reasons for the decrease in the proportion of anaerobic glycolysis are mainly related to changes in the size, number, and volume of mitochondria (Holloszy and Coyle, 1984) and an increase in capillary density (Anderson and Hendriksson, 1977; Saltin and Rowell, 1980). Exercise-induced increases in the number of mitochondria facilitates an increase in the body’s reliance on fat oxidation, decrease the proportion of glycolysis, and reduce lactate production (Egan and Zierath, 2013). Furthermore, an increase in capillary density is accompanied by an increase in the body’s ability to supply oxygen, thereby reducing blood lactate production. But there was still controversy regarding the effects on mitochondria and capillaries in HIIT and MICT (Høier et al., 2013; Cocks et al., 2015; Martins et al., 2016) which prevented us from exploring further.

The transport and clearance of blood lactate are related to the transport capacity of the circulatory system and the density of capillaries, which increases the outflow of H^+^ and blood lactate (Holloszy and Coyle, 1984). For lactate clearance, individuals with high aerobic capacity can use less energy to remove H^+^ and lactate from the body during recovery, facilitating recovery (Tomlin and Wenger, 2001; Messonnier et al., 2013). From Table 1 and Figure 1, there is no difference in peak blood lactate values between the HIIT and MICT groups. However, the 15-min and 30-min blood lactate values in the HIIT group were significantly lower than those in the MICT group, and the percentage of blood lactate clearance was significantly higher than that in the MICT group. Therefore, the lower blood lactate values in the HIIT group can be attributed to the body’s higher capacity for blood lactate transport and clearance.

It can be concluded that HIIT was more effective than MICT in the transport and clearance of blood lactate. Since greater disruption of homeostasis by exercise leads to a greater impact on restoring metabolism (Brehm and Gutin, 1986), we hypothesize that individuals who participate in HIIT over long periods can adapt to the acidic environment caused by lactate. They may increase their reserves of alkaline substances, allowing for quicker elimination of exercise-induced increases in blood lactate (Grgic et al., 2021). Previously, Gorostiaga et al. (1991) compared the effects of 8 weeks of continuous training (CT, exercise at 50% maximal work) and interval training (IT, exercise at 100% maximal work) on blood lactate clearance capacity, which is similar to the present study, however, the intensity of CT was lower than that of MICT. Although this study did not further explore the underlying mechanisms of the findings, but the results indicated that the intervention impact of HIIT surpassed that of MICT in improving blood lactate clearance following high-intensity test, with the disparity being more pronounced after 8 weeks compared to 4 weeks.

The HIIT group had a similar promotion effect to the MICT group in terms of vital capacity post-test indicators and this is consistent with previous studies (Martins et al., 2016; Su et al., 2019), which suggests that individuals can choose the appropriate workout to enhance vital capacity and reduce body fat percentage, based on their own preferences as well as their physical condition.

Certainly, besides running, there are other types of exercise as well, Wakayoshi et al. (1993) investigated the effects of aerobic swimming training on blood lactate concentration following high-intensity test. The results indicated a significant reduction in blood lactate levels after the intervention, which is consistent with the reduction in blood lactate response observed in the present study. This result shows that improving aerobic capacity lowers lactate response after high-intensity exercise and that various exercise forms can have similar effects. Furthermore, McMaster et al. (1989) found that swimming at 65% of maximal speed after high-intensity swimming was more effective in clearing blood lactate than passive rest. This underscores the importance of selecting appropriate training methods and recovery strategies.

## 5 Study limitations and future directions

As this study tested only adult males and considering that sex is also an important factor influencing the intervention (Schmitz et al., 2020; Hottenrott et al., 2021), the postintervention differences by sex could be further explored. Furthermore, as the interval duration of HIIT (Sijie et al., 2012) affects recovery, further investigation of the effect of HIIT with different intervals on lactate clearance capacity is necessary in the future. Finally, the subjects’ diet is also an important factor in lactate clearance (Wang et al., 2019), the effectiveness of lactate clearance in the body can also be investigated with both diet and exercise interventions.

## 6 Conclusion

The results of this study show that HIIT and MICT are both effective in promoting blood lactate clearance after high-intensity test, reducing body fat and increasing vital capacity in healthy adult males, with HIIT being more effective in promoting blood lactate clearance and vital capacity. Furthermore, we do not deny the role of MICT in improving blood lactate clearance, as HIIT is not suitable for everyone. Therefore, MICT remains a valuable option, particularly for individuals who require a less intense regimen or are new to exercise. Future research should focus on the integration of these training methods into personalized fitness plans and explore their benefits across different populations to optimize exercise recommendations and improve public health outcomes.

## Data Availability

The original contributions presented in the study are included in the article/Supplementary Material, further inquiries can be directed to the corresponding author.
