# Corrigendum: Effect of high-intensity interval training and moderate-intensity continuous training on blood lactate clearance after high-intensity test in adult men

**DOI:** 10.3389/fphys.2024.1505723

**Published:** 2024-10-14

**Authors:** Han Xie, Xiaojin Mao, Zhaohong Wang

**Affiliations:** ^1^ College of Physical Education and Sports, Beijing Normal University, Beijing, China; ^2^ College of Physical Education, Shandong Normal University, Jinan, China

**Keywords:** HIIT, MICT, blood lactate, adult men, train

In the published article, there was an error in the **Funding** statement. The name of the funder and the grant number were incorrectly written as “China Key projects of the National Social Science Foundation (220110269).” The correct statement appears below.

